# Intracerebroventricular Ghrelin Administration Increases Depressive-Like Behavior in Male Juvenile Rats

**DOI:** 10.3389/fnbeh.2019.00077

**Published:** 2019-04-16

**Authors:** Thomas M. Jackson, Tim D. Ostrowski, David S. Middlemas

**Affiliations:** ^1^Department of Pharmacology, Kirksville College of Osteopathic Medicine, A.T. Still University of Health Sciences, Kirksville, MO, United States; ^2^Department of Physiology, Kirksville College of Osteopathic Medicine, A.T. Still University of Health Sciences, Kirksville, MO, United States

**Keywords:** depression, ghrelin, intracerebroventricular, neurogenesis, juvenile

## Abstract

Major depressive disorder (MDD) is arguably the largest contributor to the global disease and disability burden, but very few treatment options exist for juvenile MDD patients. Ghrelin is the principal hunger-stimulating peptide, and it has also been shown to reduce depressive-like symptoms in adult rodents. We examined the effects of intracerebroventricular (icv) injection of ghrelin on depressive-like behavior. Moreover, we determined whether ghrelin increased neurogenesis in the hippocampus. Ghrelin (0.2-nM, 0.5-nM, and 1.0-nM) was administered acutely by icv injection to juvenile rats to determine the most effective dose (0.5-nM) by a validated feeding behavior test and using the forced swim test (FST) as an indicator of depressive-like behavior. 0.5-nM ghrelin was then administered icv against an artificial cerebrospinal fluid (aCSF) vehicle control to determine behavioral changes in the tail suspension test (TST) as an indicator of depressive-like behavior. Neurogenesis was investigated using a mitogenic paradigm, as well as a neurogenic paradigm to assess whether ghrelin altered neurogenesis. Newborn hippocampal cells were marked using 5′-bromo-2′-deoxyuridine (BrdU) administered intraperitoneally (ip) at either the end or the beginning of the experiment for the mitogenic and neurogenic paradigms, respectively. We found that ghrelin administration increased immobility time in the TST. Treatment with ghrelin did not change mitogenesis or neurogenesis. These results suggest that ghrelin administration does not have an antidepressant effect in juvenile rats. In contrast to adult rodents, ghrelin increases depressive-like behavior in male juvenile rats. These results highlight the need to better delineate differences in the neuropharmacology of depressive-like behavior between juvenile and adult rodents.

## Introduction

Juveniles with depression manifest a different array of symptoms than adults, which is one significant reason that major depressive disorder (MDD) may be underdiagnosed in juveniles (Yorbik et al., 2004). It has been reported that approximately 2.5% of children and 9.8% of adolescents are diagnosed with MDD. Moreover, selective serotonin reuptake inhibitors have efficacy in juvenile patients, whereas other classes of antidepressant drugs do not have demonstrated efficacy in juvenile patients (Hazell et al., 1995, 2002). The neuropharmacology of antidepressant drugs is clearly different in juvenile and adult patients.

Ghrelin is a peptide hormone that has been found to have an acute antidepressant effect in adult rodents (Carlini et al., 2012), which is correlated with an increase of hippocampal neurogenesis (Moon et al., 2009). In humans, some weak antidepressive effects of ghrelin in male depressed patients were reported (Kluge et al., 2011). Neurogenesis is a potential mechanism that could be involved in antidepressant drug action as antidepressant drugs have been shown to increase neurogenesis in adult rodents (Malberg et al., 2000). In adult rodents, neurogenesis is required for some of the antidepressant effects of antidepressant drugs such as imipramine and fluoxetine (Santarelli et al., 2003). Antidepressant drugs increase neurogenesis in the hippocampus of nonhuman primates (Perera et al., 2007). Also, an increase in the rate of hippocampal neurogenesis was observed in human progenitor cells treated with antidepressant drugs (Belmaker and Agam, 2008; Anacker et al., 2011).

Ghrelin has been recognized as the most potent endogenous stimulant of the hunger sensation (Bali and Jaggi, 2016). Recent studies have linked ghrelin to the etiology of depression, as treatment with ghrelin has been shown to decrease depressive-like symptoms related to anhedonia (Wang et al., 2015). Acute ghrelin administration has also been shown to reverse depressive-like behavior in adult rodents (Carlini et al., 2012). The receptors for ghrelin, the type 1a growth hormone secretagogue receptors, have been discovered to exist not only in the hypothalamus and pituitary, but also in the entire hippocampus (Hornsby et al., 2016). Treatment with ghrelin in adult rodents increased neuronal proliferation in the hippocampus by 50% (Moon et al., 2009).

Only selective serotonin reuptake inhibitors (SSRIs; one of five drug classes approved for treatment of depression in adults) have been demonstrated effective and only two are approved by the FDA to treat juvenile MDD. Considering the lack of antidepressant drugs available for juveniles, this study aimed to determine whether the brain-gut peptide ghrelin effectively reduces depressive-like symptoms in juvenile rats, as it does acutely in adult rodents (Carlini et al., 2012; Liu et al., 2015). In this study, we used behavioral testing to examine any changes in depressive-like behavior with the treatment of ghrelin. We also quantified hippocampal mitogenesis and neurogenesis to determine whether ghrelin influences the juvenile nervous system in the same way as the adult system.

## Materials and Methods

### Experimental Design

Male juvenile Sprague-Dawley rats post-natal day (PND < 18) were housed with their mothers in cages under regulated temperature (21 ± 1°C), humidity (46%), and light conditions (12 h light/dark cycle) at A.T. Still University’s Kirksville College of Osteopathic Medicine (KCOM). Rats age PND 18–36 were housed individually. All care and procedures involving rats were approved by the KCOM Institution Animal Care and Use Committee (IACUC). The KCOM animal care program is accredited by the Association for Assessment and Accreditation for Laboratory Animal Care (AAALAC), has an assurance (Assurance Number A3058-01) with the Office of Laboratory Animal Welfare (OLAW), and has a license (Customer No. 1495, Registration No. 43-R-0012) from the U.S. Department of Agriculture (USDA). The KCOM animal care program also has an Occupational Health and Safety Program (OHSP) supervised by the IACUC.

The first set of rats (*n* = 24) was randomly divided into an artificial cerebrospinal fluid (aCSF) treated group (*n* = 6) and 200-pmol (*n* = 6), 500-pmol (*n* = 6), and 1-nmol (*n* = 6) ghrelin treated groups. All groups underwent the forced swim test (FST) at the end of the treatment cycles (PND 29) to assess the effects of the treatments on depressive-like behavior (Katz, 1982; Martínez-Mota et al., 2011). The second set of rats (*n* = 16) was divided into a fluoxetine (5-mg/kg) treatment group and a phosphate-buffered saline (PBS) control group. These rats were subjected to the tail suspension test (TST) to determine if the TST could successfully be used in juvenile rat studies. The third set of rats (*n* = 32) was randomly divided into a mitogenic paradigm group (*n* = 16) and a neurogenic paradigm group (*n* = 16). Each of these groups was further divided into an aCSF treated group (*n* = 8) and a ghrelin treated group (*n* = 8).

**Figure 1:**
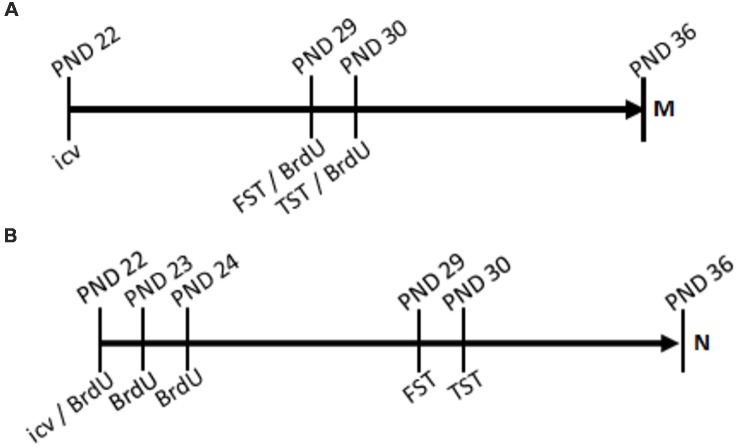
Timelines. Treatment of ghrelin or artificial cerebrospinal fluid (aCSF) control was administered by intracerebroventricular (icv) injection acutely at post-natal day 22 (PND 22). In the mitogenic paradigm **(A)**, 5′-bromo-2′-deoxyuridine (BrdU) was administered by intraperitoneal (ip) injection three times daily for two consecutive days, PND29 and PND30. In the neurogenic paradigm **(B)**, BrdU was administered twice daily for three consecutive days, PND22, PND23, and PND24.

For the last set of rats, behavioral assessments were conducted 6 and 7 days prior to euthanasia. Each group underwent the FST (PND, 29) and the TST (PND 30; timelines, Figure 1) to assess the effects of the treatments. The mitogenic group received 5′-bromo-2′-deoxyuridine (BrdU) injections three times daily for 2 days, beginning the first day of behavioral assessment, as part of the preparation for newborn cell visualization. The neurogenic group received BrdU injections twice daily for 3 days, beginning the day of drug treatment, in preparation to visualize newborn neurons. Euthanasia occurred at PND 36. The rats were anesthetized with sodium pentobarbital, then euthanized by thoracotomy, jugular exsanguination, and transcardial perfusion. Death was confirmed by respiratory and cardiac system cessations. At PND 36, the brains were removed and frozen; then, 30 μm slices were made using a cryostat (Leica CM 1900). Immunohistochemistry of brain slices was performed using primary antibodies for BrdU (for both mitogenic and neurogenic paradigms) and NeuN (for the neurogenic paradigm), with fluorescently labeled secondary antibodies excited at 594 and 488-nm, respectively. Brain slices were examined using a confocal microscope to identify differences in the number of BrdU positive cells in the hippocampal dentate gyrus for the mitogenic paradigm group and differences in colocalization of BrdU and NeuN in the neurogenic paradigm group.

### Ghrelin Administration Protocol

Acyl-ghrelin (0.2-nM, 0.5-nM, and 1.0-nM; Abbiotec, San Diego, CA, USA) dissolved in (aCSF: 123-mM NaCl, 1.14-mM CaCl_2_, 3.03-mM KCl, 1.90 -mM MgCl_2_, 25.0-mM NaHCO_3_, 0.50-mM NaH_2_PO_4_, 0.25-gmM Na_2_HPO_4_) was administered by icv injection via stereotaxic surgery. Rats (PND 21) were fasted overnight prior to surgery (for the dose response experiment), and they were anesthetized with isoflurane (5%; Piramal Group, Mumbai, India) prior to surgery (PND 22; Figure 1). Dexamethasone (2-mg/kg, s.q.; AuroMedics Pharma, LLC, Dayton, NJ, USA) was administered to the rats to prevent immunological responses that could cause the brain to swell. The rats were then placed in a stereotaxic frame (David Kopf Instruments, Tujunga, CA, USA) with ear bars to hold the head in a stable position, and isoflurane was reduced to 2% for maintenance of anesthetization. The skull was exposed, and, using an atlas of stereotaxic coordinates (Khazipov et al., 2015) and a p21 juvenile rat brain map, the lateral ventricles were located at 0.9-mm posterior and 1.5-mm lateral (on each side) from Bregma, and 3.6-mm deep from the surface of the brain. This was the site of injection with ghrelin or aCSF (control). Following closure of the incision, rats were injected with baytril (7-mg/kg, i.m.; Norbrook Laboratories, Down, Northern Ireland) to prevent infection, buprenorphine (50-μg/kg, s.q.; Reckitt Benckiser Pharmaceuticals, Richmond, VA, USA) to manage pain, and saline (2-mL, 0.9%, s.q.; Hospira, Inc., Lake Forest, IL, USA) to restore physiologic fluid balance (DeVos and Miller, 2013). Treatment occurred on PND 22, and the rats were caged individually and allowed to recover until PND 29, when they began to undergo behavioral assessment.

### Feeding Behavior Test

Food was withheld for 8 h prior to ghrelin injection. Thirty minutes after recovery from anesthesia, all animals had food available *ad libitum*. Food intake was the difference between the weight of food given and the food left prior by weight after a 2 h period.

### Forced Swim Test

A modified version of the FST was used to determine the behavioral effect of each injection and therefore the effectiveness of the target agent (acyl-ghrelin) as a potential antidepressant (Lucki, 1997; Porsolt et al., 1977; Borsini and Meli, 1988). Rats underwent the FST 1 week after the treatment with ghrelin, on PND 29 (1 week before euthanasia). Behaviors were assessed in the FST to determine each rat’s change in behavior following treatment. The efficacy of the interventions was demonstrated by an increase in the time spent swimming and/or climbing and a decrease in time spent immobile (Reed et al., 2008) compared to the aCSF vehicle control group. Swimming behavior was characterized by actively maneuvering around the container. Climbing behavior was characterized by moving the forepaws against the wall of the container in an effort to escape. Immobility behavior was characterized by floating and only making the movements necessary to keep the head above water and prevent drowning. An increase in the time spent immobile (lack of physical effort to survive) was an indicator of hopelessness, a behavioral symptom that is characteristic of a depressive state. An increase in the time spent immobile was an indicator of depressive-like behavior, as this was indicative of the characteristic depressive symptom of hopelessness (lack of physical effort to survive).

### Fluoxetine Protocol

Fluoxetine (5-mg/kg; Sigma Aldrich, St. Louis, MO, USA) dissolved in PBS was administered by intraperitoneal (ip) injection to the hypogastric region. Fluoxetine is one of only two antidepressant drugs (the other being escitalopram) that is FDA approved for treatment of depression in juvenile patients. Behavioral tests with fluoxetine were used as a positive control for observing an antidepressant effect.

### Tail Suspension Test

On PND 30 (1 day after the FST, and 6 days before euthanasia), we performed the TST. In the TST, we employed a short-term stressor—suspension by the tail via surgical tape—to assess the long-term behavioral effect of the administered ghrelin. Suspension by the tail occurred for 6 min at a time, and any struggle by the rat to escape was considered unperturbed, “non-depressed” behavior. Attempts to escape were characterized by the rat moving its feet as if to run away, attempting to climb its tail to the site of suspension, twisting and swinging to try and free itself from the tape, and scratching or biting the tape to try and free its tail. Immobility—when the rat hung limply, making no effort to escape the stressor—was considered depressive-like behavior (Steru et al., 1985). Immobility time was counted visually in 1-min intervals over the course of the 6-min test. An increase in time spent immobile was indicative of the depressive-like symptom of anhedonia, or a state of hopelessness (Carlini et al., 2012). The effectiveness of the intervention was assessed as less time spent immobile by the ghrelin (or fluoxetine)-treated group than by the aCSF (or PBS) control group.

### BrdU Administration Protocol

BrdU is a thymidine analogue that incorporates into the DNA during the synthesis phase of the cell cycle. Thus, it marks newborn cells and is used as a label for mitogenesis (Taupin, 2007). Rats received i.p. injections of BrdU (Sigma-Aldrich, St. Louis, MO, USA) dissolved in PBS (11.9 mM phosphates, 137 mM sodium chloride, 2.7 mM potassium chloride; Fischer Scientific, Houston, TX, USA; Wojtowicz and Kee, 2006). These injections were administered at a fixed volume of 5 μL/g three times daily for two consecutive days, beginning the day of the FST (PND 29–30) for the mitogenic paradigm group, and for the neurogenic paradigm group, rats received the BrdU injections at a volume of 2.5 μL/g twice daily for three consecutive days, beginning the day of drug treatment (PND 22–24; Figure 1). Since the process of neurogenesis takes approximately 5 to 7 days to occur, the neurogenic group received BrdU earlier in the treatment schedule so that the neurons visualized would be the differentiated newborn cells whose DNA was traced by BrdU upon proliferation.

### Transcardial Perfusion

The brains of the rats were collected using the transcardial perfusion protocol as described by Wojtowicz and Kee (2006). The rats were anesthetized by IP injection of 50 mg/kg of sodium pentobarbital (PND 36). We determined complete anesthetization by observing corneal, foot withdrawal, and tail squeeze reflexes. Once complete anesthetization was determined, the rats were then sacrificed. The thoracic wall was bluntly dissected to expose the heart. A needle was injected into the left ventricle, and the jugular veins were severed to allow an outflow of fluids. The brains were then flushed via transcardial perfusion with 35 mL of PBS. Then, the brains were perfusion-fixed with 35 mL of 4% paraformaldehyde in PBS. Once the brains were removed, they were weighed and subsequently placed in 4% paraformaldehyde for 24 h. We then transferred the brains to a vial of 30% sucrose and PBS solution for 3 days or until they sank to the bottom of the vial (van Praag et al., 2005). Finally, the brains were frozen by placing them on dry ice (Sartori et al., 2004) and then were stored at −80°C.

### Stereotaxic Slicing Protocol

Whole brains were sliced into 30 μm coronal sections using a cryostat (Leica CM 1900) set at a temperature of −22°C. The brains were sliced through the dentate gyrus of the hippocampus. Prior to slicing, we used the Atlas of the Postnatal Rat Brain in Stereotaxic Coordinates (Khazipov et al., 2015) for orientation purposes. A total of 96 slices were obtained for each rat brain, slices were stored at 4°C in PBS with 0.1% sodium azide, and every 12th slice was used for immunohistochemistry (eight slices).

### Immunohistochemistry

Newly differentiated (newborn) neurons in the dentate gyrus of the dorsal hippocampus were identified by immunohistochemistry. Fluorescent antibodies allowed us to visualize cells containing BrdU and NeuN (a neuronal nuclear marker; Wojtowicz and Kee, 2006). BrdU was administered to mark newborn cells; NeuN is an endogenous protein present in neuronal nuclei. The assay for BrdU and NeuN allowed for the visualization of newborn cells and those that had differentiated into neurons. The brain slices were washed in PBS three times for 5 min each. Brain slices were then incubated in 1-M hydrochloric acid at 45°C for 1 h. Then, the brain slices were washed six times, for 5 min each, with PBS and incubated in a blocking solution (PBS, 0.3% Triton X-100, 2% equine serum; Invitrogen Corporation, Carlsbad, CA, USA) for 1 h. Once the blocking solution was removed, the brain slices were placed in a 1:4,000 solution of primary antibody (rat anti-BrdU, Batch No. 1015, Bio-Rad Laboratories, Hercules, CA, USA; mouse anti-NeuN, Lot #2950736, EMD Millipore Corporation, Temecula, CA, USA) in blocking solution. The slices were then incubated for 24 h at 4°C. Brain slices were washed three times for 5 min each with PBS, and they were then incubated with secondary antibodies (Alexa Fluor 594 donkey anti-rat, Lot# 1870948, Invitrogen, Carlsbad, CA, USA; Alexa Fluor 488 goat anti-mouse, Lot #1834337, Invitrogen, Carlsbad, CA, USA) diluted at 1:1,000 in PBS with 0.3% Triton x-100 for 2 h. Slices were washed three times for 5 min each in PBS and then mounted with Permount Toluene Solution (Lot #155508, Fisher Scientific, Waltham, MA, USA) onto microscope slides.

### Confocal Microscopic Imaging

Newborn neuron survival was assessed using confocal microscopy (Leica DMI 6000B confocal laser scanning microscope). Colocalization of the signal from BrdU and NeuN constituted neurogenesis. We analyzed colocalized fluorescence using the Leica LAS Advanced Fluorescent software. Visual thresholds were set for BrdU^+^ (red) and NeuN^+^ (green) signals; the overlap two signals (white), represented cells positive both for BrdU and NeuN.

### Data Analysis

One-way ANOVA tests with Tukey post-tests were used to determine whether there was a difference in the feeding behavior test and the FST. To determine whether a change in immobility time was correlated with a change in mitogenesis or neurogenesis, a two-tailed Pearson product-moment correlation coefficient was performed. Analysis of all experiments comparing aCSF control and ghrelin treatment groups was performed using an unpaired two-tailed *t*-test. *P*-values < 0.05 were considered significant. Data were analyzed using GraphPad Prism software.

## Results

### Tail Suspension Test Validation

The TST is a behavioral test that has been used exclusively in mouse studies. To determine whether or not the TST could be used to measure depressive-like behavior in juvenile rats, we used fluoxetine as a positive control; fluoxetine is one of two FDA- approved antidepressant treatments for human juveniles. It was found that the fluoxetine-treated group spent less time immobile in the TST than the saline control group (data not shown, *p* < 0.05, Mean ± SD; control 92 ± 1.2, Fluoxetine 71 ± 7.2). Showing that fluoxetine produced a measurable effect validated that the TST can be used to assess depressive-like behavior in juvenile rats. This was contrasted to Fluoxetine in the FST in juvenile rats (*p* > 0.05, Mean ± SD; control 18 ± 32, fluoxetine 7 ± 8). This led to using the TST in the mitogenic and neurogenic experiments, as the TST has less variability in young adolescent rats then the FST.

### Intracerebroventricular Injection Validation

**Figure 2:**
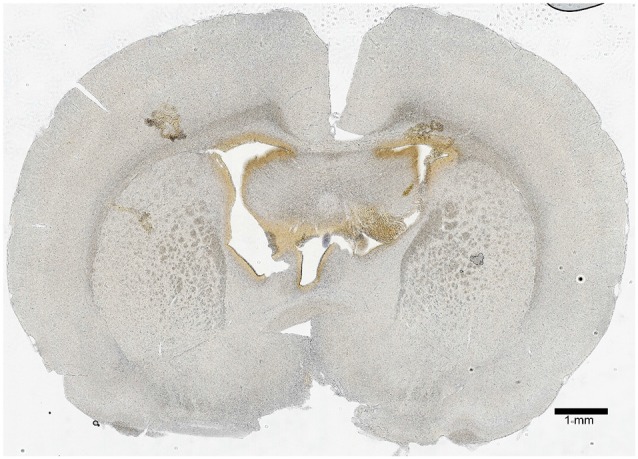
Icv injection validation. A 50-μm coronal section 0.9-mm posterior from Bregma was imaged to show the site of icv injection. The lateral ventricles were located 1.5-mm lateral from Bregma (on both sides), and the injection was made at a depth of 3.6-mm from the surface of the brain. The hole-like structures in the central region of the section show the ventricle system, and the brown coloration is residue from a tryptophan blue control injection. The scale bar represents a length of 1-mm of brain tissue.

A PND 21 brain map (Paxinos and Watson) was used initially to approximate the location of the lateral ventricles; then, the surgery was performed with injection of tryptophan blue in aCSF to visualize the locations of injection (brown stain; Figure 2). It was determined that the proper stereotaxic coordinates for icv injection of PND 22 rats were 0.9-mm posterior and 1.5-mm lateral (both sides) from Bregma, and 3.6-mm deep from the surface of the brain.

### Ghrelin Dose-Response on a Feeding Behavior and the Forced Swim Tests

**Figure 3:**
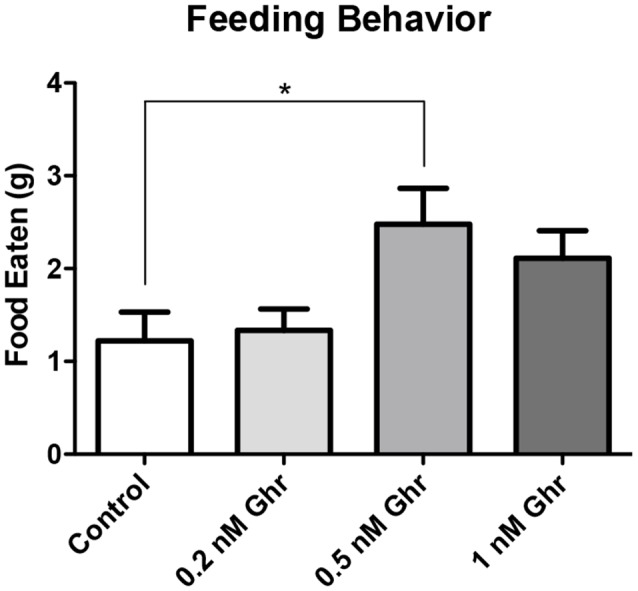
Dose response: feeding behavior test. Weight of food consumed within 2 h following recovery from anesthetization (PND2). Values expressed as mean + SEM (one way ANOVA, *p* = 0.02, Tukey’s Multiple Comparison Test, *p < 0.05).

**Figure 4:**
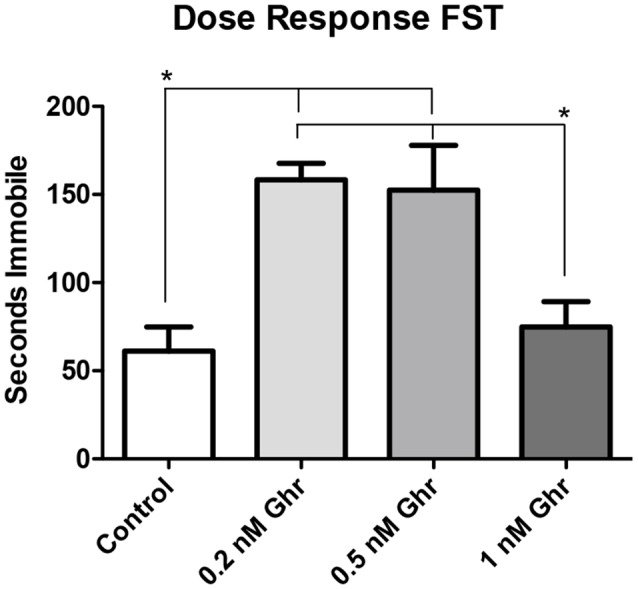
Dose response: forced swim test (FST). Time spent immobile in the FST. Values expressed as mean + SEM (one way ANOVA, *p* < 0.01), Tukey’s Multiple Comparison Test, *p < 0.05.

To determine the most effective dose of ghrelin, a feeding behavior test and the FST were performed. Four groups were treated with 0, 0.2, 0.5-nM, and 1 nM ghrelin respectively (Figure 3, *p* = 0.02). From this result, it was determined that the 0.5-nM concentration was the most effective dose of acyl-ghrelin for eliciting the hunger sensation. In the FST, the 0.2-nM (*p* < 0.0001) and 0.5-nM (*p* = 0.008) groups demonstrated significant increases in immobility time (Figure 4). Contrary to adult rodents, treatment with ghrelin in juvenile rats did not yield changes indicative of an antidepressant-like effect.

Also, there was no significant difference in both feeding behavior and immobility time in the FST among the 1-nM group; perhaps this was due to desensitization of ghrelin receptors. Therefore, the 0.5-nM concentration was used throughout the remainder of the experiments.

**Figure 5:**
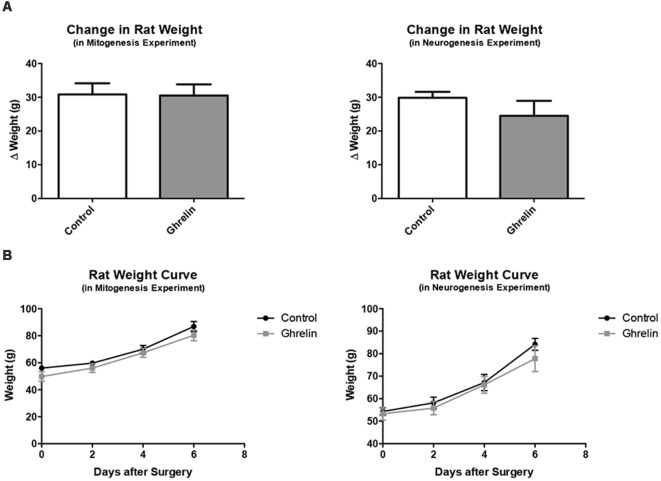
Rat weights for mitogenesis experiment. **(A)** For the mitogenesis and neurogenesis experiments, we compared the change in weights from the day of surgery (PND 22) to 1 week following surgery (PND 29) for both the control (*n* = 6) and ghrelin treated (*n* = 6) groups **(A)**. Values are expressed as mean ± SEM (*p* > 0.05). **(B)** We also compared the weights of control and ghrelin-treated groups every 2 days from the day of surgery to 1 week following surgery (two way repeated measures ANOVA, *p* > 0.05).

To assess whether injection of ghrelin had a lasting effect on feeding behavior, the change in weight of the juvenile rats from the day of icv injection surgery (PND 22) to the day of behavioral testing (PND 29) was compared between the control and ghrelin treated groups (Figure 5). Also, curves were generated to depict the progression of rat weights every 2 days from PND 22 to PND 29 (Figure 5). It was found that there was no significant difference between control and ghrelin (*p* = 0.4724) in terms of weight change. Also, the weight curves were virtually parallel, indicating that the only difference between ghrelin and control rats was the average weights per group, a difference consequent of randomized placement into groups. This analysis demonstrated that the acute effect of ghrelin—the stimulation of the hunger sensation and therefore the drive to consume food—did not persist beyond the time period between icv injection and the end of the feeding behavior test, and furthermore did not affect long-term feeding behavior of the rats. Also, the half-life of acyl-ghrelin is approximately 29 min (Akamizu et al., 2004), which is yet another indication that the acute effect of ghrelin would not be present beyond the day of administration.

### TST and Mitogenesis

**Figure 6:**
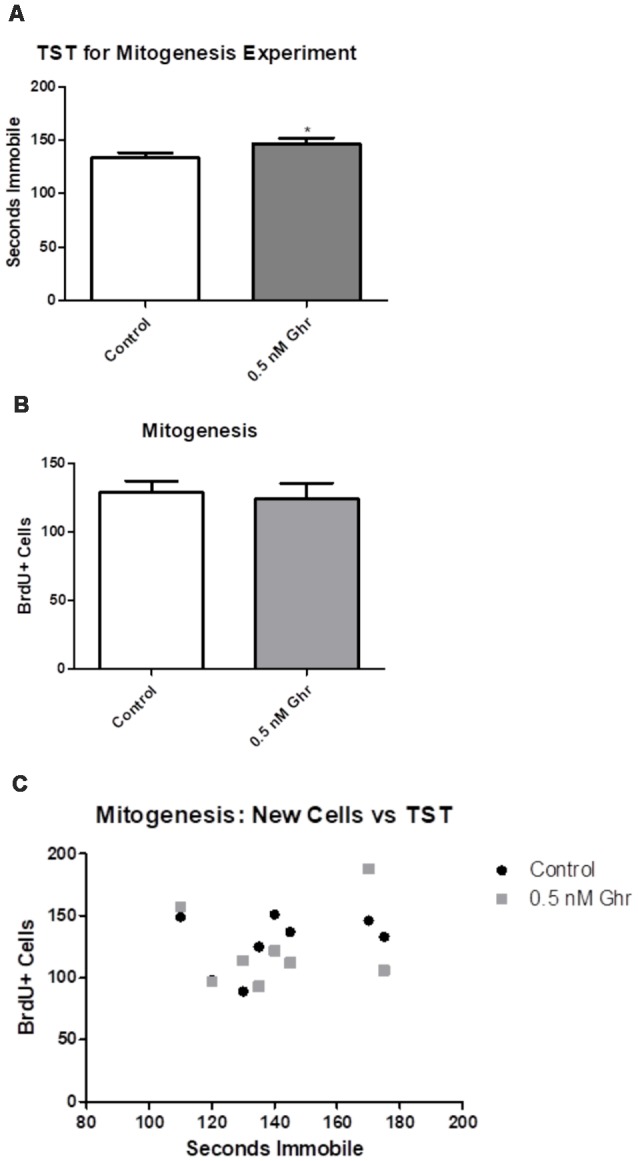
Mitogenesis and the tail suspension test (TST). Behavioral tests as conducted for the mitogenic paradigm. **(A)** TST for the mitogenic paradigm. Values expressed as mean + SEM (*p < 0.05). **(B)** Number of cells that contained BrdU, as quantified using confocal microscopy. Values expressed as mean + SEM (*p < 0.05). **(C)** Linear comparison of the TST to the number of BrdU positive cells.

To quantify any changes in the proliferation of neural stem cells in the dorsal hippocampal dentate gyrus, BrdU was injected 7 and 8 days after ghrelin administration to determine if ghrelin altered the basal mitogenic rate (Figure 1). We performed immunohistochemistry and visualized coronal sections of rat brains using confocal microscopy. Cells positive for BrdU (newly proliferated neural stem cells) appeared red; cells positive for NeuN (neurons) appeared green; with the overlay of the two signals, cells that were positive for both BrdU and NeuN (newborn neurons) appeared white. The TST showed that ghrelin-treated rats spent more time immobile than aCSF vehicle-treated rats (*p* = 0.04; Figure 6A). There was no significant difference in mitogenesis (quantity of newly formed cells in the hippocampal dentate gyrus) between the control and 0.5-nM ghrelin treatment groups (*p* = 0.74; Figure 6B). There was also no correlation with a linear regression line between the TST immobility time and mitogenesis (Figure 6C; control *p* = 0.46, ghrelin *p* = 0.63). These results demonstrated that ghrelin did not increase hippocampal mitogenesis; so, treatment with ghrelin did not yield changes indicative of an antidepressant-like effect.

### TST and Neurogenesis

**Figure 7:**
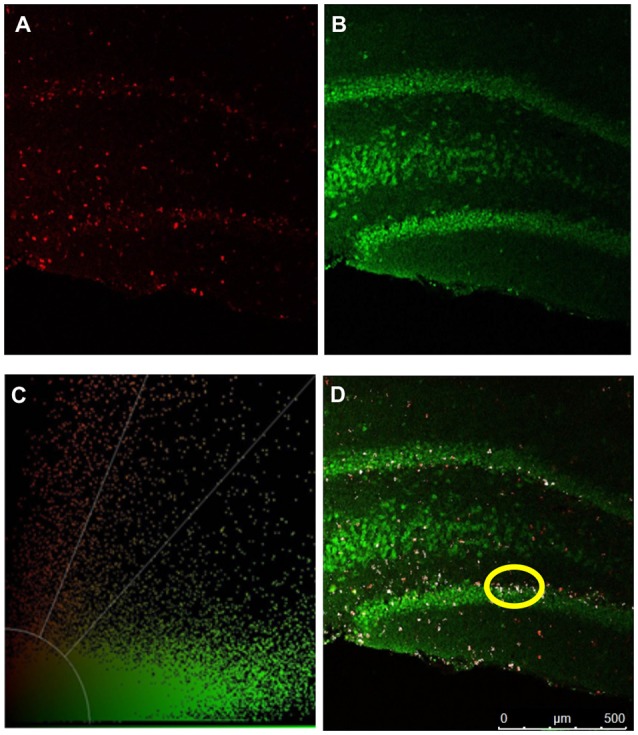
Confocal microscopy. Confocal microscopy was used to quantify mitogenesis and neurogenesis in the hippocampal dentate gyrus. IHC detection of BrdU and NeuN are depicted in pseudocolored images. **(A)** BrdU positive cells (red). **(B)** NeuN positive (green) cells. **(C)** Threshold of color intensities. **(D)** An overlap of **(A,B)**. Cells that have colocalized signal from both BrdU labeling and NeuN expression are depicted in white (example circled in yellow).

**Figure 8:**
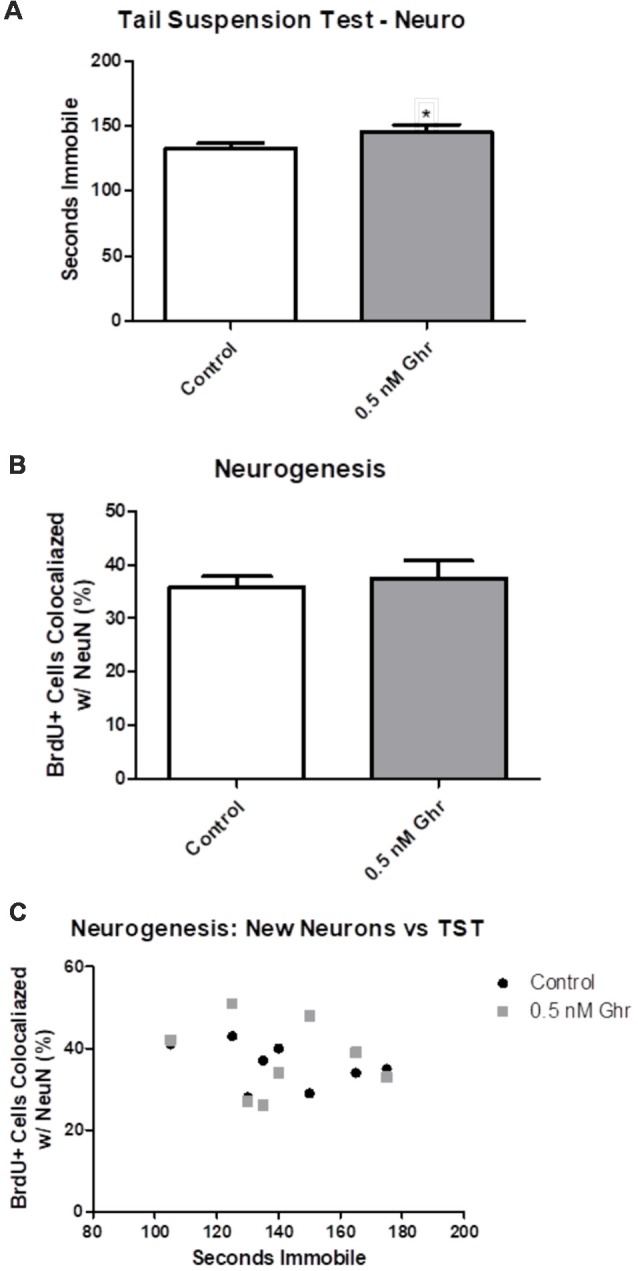
Neurogenesis and the TST. **(A)** TST for the neurogenic paradigm. **(B)** Number of cells that contained both BrdU and NeuN, as quantified using confocal microscopy. **(C)** Linear comparison of the TST to the percentage of BrdU positive cells that were co-localized with NeuN. Values expressed as mean + SEM (*p < 0.05).

To quantify any changes in neurogenesis in the hippocampal dentate gyrus, BrdU was injected initially in the neurogenic paradigm to assess if there was a difference in neurogenesis (Figure 1). We performed immunohistochemistry and visualized coronal brain sections using confocal microscopy (Figure 7); we also assessed behavior in the TST. The TST showed that ghrelin-treated rats spent more time immobile than the aCSF vehicle-treated rats (*p* = 0.04; Figure 8A). There was no significant difference in the neurogenic ratio (percentage of newly formed hippocampal cells that differentiated into neurons) between the control and 0.5-nM ghrelin treatment groups (Figure 8B; *p* = 0.67). There was also no correlation with a linear regression line between the TST immobility time and neurogenesis (Figure 8C; control *p* = 0.30, ghrelin *p* = 0.73). These results indicated that treatment with ghrelin did not yield changes in the hippocampus indicative of an antidepressant-like effect.

## Discussion

Ghrelin is a hunger-stimulating brain-gut peptide that has been implicated as a potential antidepressant in studies involving adult rodents. In adult rodent studies, acute treatment with ghrelin was shown to decrease depressive-like behavior, indicated by decreases in immobility time in behavioral tests. Treatment with ghrelin was also shown to increase hippocampal mitogenesis (neural precursor cell proliferation) and neurogenesis in adult rodents. It is hypothesized that immobility time in the FST mimics the depressive-like behaviors of despondency and anhedonia (Marks et al., 2009). However, we found that acute icv administration of ghrelin increased depressive-like behavior in male, juvenile rats using the FST. Moreover, this finding was reproduced using the TST, that icv administration of ghrelin increases depressive-like behavior in male juvenile rats.

Although acute administration of ghrelin in adult rats has antidepressant effects (Carlini et al., 2012), chronic infusion over weeks increases depression-like behavior in adult rats (Hansson et al., 2011). Ghrelin is involved in activation of the HPA axis, which correlates with the increase in depressive-like behavior observed with chronic ghrelin infusion (Spencer et al., 2015). There are also other methods for chronic central administration of ghrelin (Choi et al., 2013). We have found that a single administration of ghrelin increases depressive-like behavior a week later in juvenile rats. Sustained effects like this on emotional behavior are not unprecedented. Single administration of either ketamine or brain-derived neurotrophic factor have long-lasting antidepressant effects in rats (Hoshaw et al., 2005; Browne and Lucki, 2013). It will be interesting to further explore the acute and chronic effects of ghrelin in a juvenile rat model, and especially to determine if the effects of ghrelin are long lived.

The TST revealed an increase in immobility time in the ghrelin-treated group, as compared to the control group. The TST was validated in juvenile rats using fluoxetine, which is predicted to be an antidepressant drug in juvenile rats using the FST (Yoo et al., 2013). Fluoxetine produced a significant decrease in depressive-like behavior in the TST. As an increase in immobility time in the TST is an indicator of increased depressive-like behavior (Cryan et al., 2005), these experiments demonstrated that treatment with ghrelin increases depressive-like behavior in juvenile rats. This is contrary to the effect of ghrelin in adult rodents, where it has been shown to decrease depressive-like behavior (Carlini et al., 2012; Wang et al., 2015). These differences may be attributable to developmental differences in neural pathways related to the mechanism of action of antidepressant drugs. The norepinephrine system, for example, is not fully developed in rats until sexual maturation is achieved—at about PND 49 (Bylund and Reed, 2007; Sengupta, 2013). The norepinephrine system is necessary in the mechanism of action of nearly all classes of antidepressants—the serotonin-norepinephrine reuptake inhibitors, the tricyclic antidepressants, the monoamine oxidase inhibitors, and the atypical antidepressants. The only class that does not function through the noradrenergic system, the selective serotonin reuptake inhibitors, are notably the only antidepressant drugs that effectively reduce depressive symptoms in juvenile patients. Indeed, the only two antidepressant drugs that are FDA approved for treatment of juveniles, fluoxetine and escitalopram, are selective serotonin reuptake inhibitors. It is hypothesized that many antidepressant drugs work in adults but not in juveniles because the serotonergic system develops before the noradrenergic system, and most antidepressant drugs function through noradrenergic stimulation (Murrin et al., 2007). This could also explain the developmental difference seen in the effect of ghrelin on depressive-like behavior and on hippocampal mitogenesis and neurogenesis since it has been shown that many effects of ghrelin are produced through alpha-1 and beta-2 stimulation of the noradrenergic system (Date et al., 2006).

Antidepressant-like activity of drugs in adult rodents is correlated with increased hippocampal mitogenesis (David et al., 2009) and Ghrelin has been found to increased mitogenesis in adult mice (Moon et al., 2009). However, we found that treatment with ghrelin did not alter mitogenesis in the hippocampal dentate gyrus in male, juvenile rats. There was no significant difference between the control group and the ghrelin-treated group in terms of the number of new cells formed in the hippocampal dentate gyrus. Moreover, we found that there was also no significant difference between the control and ghrelin-treated groups on juvenile rat hippocampal neurogenesis. This investigation focused on the dorsal hippocampus because previous work revealed chronic fluoxetine treatment of juvenile male rats increased mitogenesis. However, there is evidence that ghrelin-mediated feeding behavior may involve the ventral hippocampus (Kanoski et al., 2013). These results are opposite the observations in adult rodent studies. Ghrelin increased mitogenesis and neurogenesis in adult rodents, but it did not affect mitogenesis or neurogenesis in juvenile rats.

Based on previous studies demonstrating that acute icv administration of ghrelin had antidepressant effects in adult rats, and moreover, increased hippocampal neurogenesis, we hypothesized that administration of acyl-ghrelin to juvenile rats would also have an antidepressant effect and increase hippocampal neurogenesis. However, our findings indicate just the opposite, that ghrelin increases depressive-like behavior in juvenile rats. This is more in line with the effects of chronic ghrelin infusion in adult rats over weeks, where ghrelin increases depression-like behavior (Hansson et al., 2011). Using the FST, ghrelin did not have an antidepressant-like effect in juvenile rats, as there was an increase in depressive-like behavior in the dose-response experiment. Also, using the TST, ghrelin was demonstrated to actually have increased depressive-like behavior in juvenile rats; that is, there was an increase in immobility time among the ghrelin-treated rats. There were no significant differences in cell proliferation or the generation of new neurons, respectively, in the hippocampal dentate gyrus. This further demonstrated that ghrelin, unlike the selective serotonin reuptake inhibitors, does not increase hippocampal neurogenesis in male, juvenile rats. It will be interesting to test whether the chronic ghrelin administration decreases hippocampal neurogenesis. These results highlight the urgent need to better understand the differences between depression in juveniles and adults in order to develop better treatment options for juvenile patients with MDD.

## Ethics Statement

All care and procedures involving rats were approved by the KCOM Institution Animal Care and Use Committee (IACUC). The KCOM animal care program is accredited by the Association for Assessment and Accreditation for Laboratory Animal Care (AAALAC), has an assurance (Assurance Number A3058-01) with the Office of Laboratory Animal Welfare (OLAW), and has a license (Customer No. 1495, Registration No. 43-R-0012) from the US Department of Agriculture (USDA). The KCOM animal care program also has an Occupational Health and Safety Program (OHSP) supervised by the IACUC.

## Author Contributions

TJ completed most of the experiments under collaborative supervision by TO and DM.

## Conflict of Interest Statement

The authors declare that the research was conducted in the absence of any commercial or financial relationships that could be construed as a potential conflict of interest.
